# Supplementary material for: Mpox knowledge and positive attitudes in Sub-Saharan African healthcare workers after 2022 outbreak of disease: A systematic review and meta-analysis
Source: PLoS Negl Trop Dis. 2026 Feb 9;20(2):e0013977. doi: 10.1371/journal.pntd.0013977 (PMC12900440; doi:10.1371/journal.pntd.0013977)
Supplement: S2 File — (PDF) [file pntd.0013977.s002.pdf]

Supplementary File 2: Full search strategies or strings

Date Range: 2022–2025

No language restrictions applied in the search syntax (though only English articles were included in the final review).

Database	Platform	Search Strategy (Core Elements)
PubMed/MEDLINE	PubMed	(monkeypox[MeSH] OR mpox) AND (knowledge[MeSH] OR attitude[MeSH]) AND (health personnel[MeSH] OR healthcare worker*) AND (Africa South of the Sahara[MeSH] OR Sub-Saharan Africa) AND 2022:2025[dp]
Embase	Elsevier	(monkeypox/exp OR mpox) AND (knowledge/exp OR attitude/exp) AND (health care personnel/exp OR healthcare worker*) AND (subsaharan africa/exp OR africa) AND [2022-2025]/py
Scopus	Elsevier	TITLE-ABS-KEY((monkeypox OR mpox) AND (knowledge OR attitude) AND ("healthcare worker*") AND ("Sub-Saharan Africa" OR Africa)) AND PUBYEAR > 2021
Web of Science	Clarivate	TS=((monkeypox OR mpox) AND (knowledge OR attitude) AND ("healthcare worker*") AND ("Sub-Saharan Africa")) AND PY=(2022-2025)
CINAHL	EBSCOhost	(MH "Monkeypox+") AND (MH "Knowledge+" OR MH "Attitude+") AND (MH "Health Personnel+") AND (MH "Africa South of the Sahara+") AND YR 2022-2025
African Index Medicus	WHO GIM	(monkeypox OR mpox) AND (knowledge OR attitude) AND ("healthcare worker*") AND (Africa OR "Sub-Saharan Africa")
Supplementary Searches	Google Scholar, Direct Google	Gray Literature: Manual search of WHO AFRO, Africa CDC, and national public health institute websites using standardized geographical terms.
