# Supplementary material for: Mpox knowledge and positive attitudes in Sub-Saharan African healthcare workers after 2022 outbreak of disease: A systematic review and meta-analysis
Source: PLoS Negl Trop Dis. 2026 Feb 9;20(2):e0013977. doi: 10.1371/journal.pntd.0013977 (PMC12900440; doi:10.1371/journal.pntd.0013977)
Supplement: S3 File — (DOCX) [file pntd.0013977.s003.docx]

| Author, year | Included (Yes/No) | Analysis included in | Reason for exclusion (if applicable) |
| --- | --- | --- | --- |
| Almaw et al, 2024 | Yes | Knowledge & Attitude |  |
| Aynalem et al, 2025 | Yes | Knowledge & Attitude |  |
| Beyna et al, 2025 | Yes | Knowledge & Attitude |  |
| Fetensa et al, 2025 | Yes | Knowledge & Attitude |  |
| Kiros et al, 2025 | Yes | Knowledge & Attitude |  |
| Abdulmumin et al ,2023 | Yes | Knowledge & Attitude |  |
| Sofonias et al, 2024 | Yes | Knowledge Only | Did not report attitude outcomes |
| Oche et al, 2024 | Yes | Knowledge Only | Did not report attitude outcomes |
| Orok et al, 2024 | Yes | Knowledge & Attitude |  |
| Ajayi et al, 2023 | Yes | Knowledge Only | Did not report attitude outcomes |
| Uche et al, 2024 | Yes | Knowledge Only | Did not report attitude outcomes |
| Iwuafor et al, 2023 | Yes | Knowledge Only | Did not report attitude outcomes |
| Epipode et al, 2025 | Yes | Knowledge Only | Did not report attitude outcomes |
| Namakula et al 2025 | Yes | Knowledge Only | Did not report attitude outcomes |
| Joyce et al 2025 | Yes | Knowledge & Attitude |  |
| Nka et al 2024 | Yes | Knowledge Only | Did not report attitude outcomes |
| [One unavailable study] | No | ---- | Could not retrieve full text |

**S3 File: Included & excluded studies table**
