# Supplementary material for: Mpox knowledge and positive attitudes in Sub-Saharan African healthcare workers after 2022 outbreak of disease: A systematic review and meta-analysis
Source: PLoS Negl Trop Dis. 2026 Feb 9;20(2):e0013977. doi: 10.1371/journal.pntd.0013977 (PMC12900440; doi:10.1371/journal.pntd.0013977)
Supplement: S5 File — (DOCX) [file pntd.0013977.s005.docx]

**S5 File: Missing data handling**

This document details the procedures used to handle missing data in the systematic review and meta-analysis titled "Mpox Knowledge and Positive Attitudes in Sub-Saharan African Healthcare Workers After 2022 Outbreak of Disease." The approach aligns with PRISMA guidelines and best practices for meta-analysis of observational studies.

**Types of Missing Data Encountered**

- **Missing outcome data** of attitude was identified during the review process of the extracted data (attitude scores not reported).

**Handling Procedures of Missing Outcomes**

- Studies reporting only knowledge outcomes (without attitude data) were included **only in the knowledge meta-analysis.**
- Studies reporting only attitude outcomes (without knowledge data) would have been included only in attitude analysis, though none were identified.

**Example:** Oche et al. (2024) reported knowledge outcomes but not attitude outcomes; therefore, it was included in knowledge analysis (n=16 studies) but excluded from attitude analysis (n=8 studies).

- **No statistical imputation** was performed for missing values or partially reported data within individual studies.
- All meta-analyses were conducted using **complete case data** as reported in the original publications.
- Where percentages were reported without corresponding counts, the counts were calculated where possible; otherwise, percentages were used directly.

**Attitude data availability disparity**

- Of the 16 studies included in knowledge analysis, only 8 reported attitude outcomes.
- This disparity was:
- Acknowledged as a limitation in the manuscript
- Considered in the interpretation of attitude results

**Statistical Analysis Implications**

- The handling of missing data influenced statistical analyses:
- **Heterogeneity estimates** (*I²* statistics) may be influenced by varying completeness of reporting across studies.
- **Subgroup analyses** were limited to countries with sufficient data (Ethiopia and Nigeria only).
- **Publication bias assessment** for attitude studies was not performed due to insufficient studies (n<10).

**Transparency and Reproducibility**

- To ensure transparency:
- All excluded studies are listed in supplementary File 3 with reasons for exclusion.
- The complete data extraction table (Supplementary File 4) indicates "NR" (Not Reported) for missing data.
- The PRISMA flow diagram (Fig 1) documents the study selection process.
