# Supplementary material for: Mpox knowledge and positive attitudes in Sub-Saharan African healthcare workers after 2022 outbreak of disease: A systematic review and meta-analysis
Source: PLoS Negl Trop Dis. 2026 Feb 9;20(2):e0013977. doi: 10.1371/journal.pntd.0013977 (PMC12900440; doi:10.1371/journal.pntd.0013977)
Supplement: Attachment — Submitted filename: PNTD-D-25-01469.pdf [file pntd.0013977.s006.pdf]

PLOS Neglected Tropical Diseases

Mpox Knowledge and Positive Attitudes in Sub-Saharan African Healthcare Workers After 2022 Outbreak of Disease: A Systematic Review and Meta-Analysis

--Manuscript Draft--

**Manuscript Number:**	PNTD-D-25-01469
**Full Title:**	Mpox Knowledge and Positive Attitudes in Sub-Saharan African Healthcare Workers After 2022 Outbreak of Disease: A Systematic Review and Meta-Analysis
**Short Title:**	Mpox Knowledge and Positive Attitudes in Sub-Saharan African Healthcare Workers
**Article Type:**	Research Article
**Keywords:**	Attitude, healthcare workers, monkeypox, Mpox, Knowledge, Sub-Saharan Africa
**Abstract:**	Background: Mpox remains a public health emergency of international concern, especially in regions beyond its usual endemic areas in Africa. Assessing healthcare providers' knowledge and attitudes is essential for effective prevention and control efforts. This systematic review and meta-analysis aimed to determine the pooled good knowledge and positive attitudes toward mpox among healthcare workers in Sub-Saharan Africa after the 2022 outbreak.<br>Methods: We have searched major databases for relevant studies published up to June 25, 2025. Studies reporting knowledge and/or attitudes towards mpox were included. The quality of the included articles was assessed using the Joanna Briggs Institute's Statistical Meta-Analysis Review Instrument, and heterogeneity was assessed using the I2 statistic. Data were extracted using a standardized protocol, and a random-effects model was used to calculate pooled prevalence estimates with 95% confidence intervals.<br>Results: The meta-analysis included 15 and 8 studies in knowledge and attitude analysis, respectively, to estimate the pooled prevalence. The pooled prevalence of good knowledge and positive attitudes towards monkeypox was 45.99% (95% CI: 36.98, 54.99) and 53.83% (95% CI: 42.96, 64.7), respectively. Significant heterogeneity was observed across studies; however, both statistical tests (Egger's test, p=0.1939; Begg's test, p=0.1982) indicated no significant publication bias excluded the presence of publication bias.<br>Conclusion: The knowledge and attitudes of healthcare workers towards monkeypox was low and unsatisfactory in sub-Saharan Africa. The review result underscores the need for targeted interventions to improve healthcare providers' understanding of mpox transmission, prevention, and management. Targeted educational programs and training are needed to improve the preparedness of healthcare workers.
**Additional Information:**	
**Question**	**Response**
**Government Employee**<br><br>Are you or any of the contributing authors an employee of the United States government?<br><br>Manuscripts authored by one or more US Government employees are not copyrighted, but are licensed under a CC0 Public Domain Dedication, which allows unlimited distribution and reuse of the article for any lawful purpose. This is a legal requirement for US Government employees.	No - No authors are employees of the U.S. government.

Powered by Editorial Manager® and ProduXion Manager® from Aries Systems Corporation

This will be typeset if the manuscript is accepted for publication.	
**Financial Disclosure**<br><br>Enter a financial disclosure statement that describes the sources of funding for the work included in this submission. Review the submission guidelines for detailed requirements. View published research articles from *PLOS Neglected Tropical Diseases* for specific examples.<br><br>This statement is required for submission and **will appear in the published article** if the submission is accepted. Please make sure it is accurate.<br><br>**Funded studies**<br>Enter a statement with the following details:<br>• Initials of the authors who received each award<br>• Grant numbers awarded to each author<br>• The full name of each funder<br>• URL of each funder website<br>• Did the sponsors or funders play any role in the study design, data collection and analysis, decision to publish, or preparation of the manuscript?<br><br>Did you receive funding for this work?	The author(s) received no specific funding for this work.
**Competing Interests**<br><br>On behalf of all authors, disclose any competing interests that could be perceived to bias this work.<br><br>This statement will be typeset if the manuscript is accepted for publication.<br><br>Review the instructions link below and PLOS NTDs' competing interests policy to determine what information must be disclosed at submission.	The authors have declared that there are no competing interests

**Data Availability**<br><br>Provide a **Data Availability Statement** in the box below. This statement should detail where the data used in this submission can be accessed. This statement will be typeset if the manuscript is accepted for publication.<br><br>Before publication, authors are required to make all data underlying their findings fully available, without restriction. Review our PLOS Data Policy page for detailed information on this policy. Instructions for writing your Data Availability statement can be accessed via the Instructions link below.	Data will be provided by the corresponding author upon reasonable request.

Manuscript
Click here to access/download;Manuscript;Mpox Manuscript SSA Plose.docx

Mpox Knowledge and Positive Attitudes in Sub-Saharan African Healthcare Workers After 2022 Outbreak of Disease: A Systematic Review and Meta-Analysis

Melaku Laikemariam[1*]

Alemayehu Molla Wollie[2]

Amare Mebrat Delie[3]

Abebe Yenesew[4]

Abateneh Melkamu[5]

Habtamu Ayele[6]

Yihalem Abeje[1]

[1]Department of Midwifery, College of Medicine and Health Sciences, Injibara University, Injibara, Ethiopia

[2]Department of Psychiatry, College of Medicine and Health Sciences, Injibara University, Injibara, Ethiopia

[3]Department of Public Health, College of Medicine and Health Sciences, Injibara University, Injibara, Ethiopia

[4]Department of Medical Laboratory science, College of Medicine and Health Sciences, Injibara University, Injibara, Ethiopia

[5]Department of Medical Laboratory science, College of Health Sciences, Debre Markos University, Debre Markos, Ethiopia

[6]Department of Midwifery, College of Health Sciences, Debre Markos University, Debre Markos, Ethiopia

*Corresponding author: Email: laikemariam2014@gmail.com (ML)

Abstract

Background: Mpox remains a public health emergency of international concern, especially in regions beyond its usual endemic areas in Africa. Assessing healthcare providers' knowledge and attitudes is essential for effective prevention and control efforts. This systematic review and meta-analysis aimed to determine the pooled good knowledge and positive attitudes toward mpox among healthcare workers in Sub-Saharan Africa after the 2022 outbreak.

Methods: We have searched major databases for relevant studies published up to June 25, 2025. Studies reporting knowledge and/or attitudes towards mpox were included. The quality of the included articles was assessed using the Joanna Briggs Institute's Statistical Meta-Analysis Review Instrument, and heterogeneity was assessed using the I^2 statistic. Data were extracted using a standardized protocol, and a random-effects model was used to calculate pooled prevalence estimates with 95% confidence intervals.

Results: The meta-analysis included 15 and 8 studies in knowledge and attitude analysis, respectively, to estimate the pooled prevalence. The pooled prevalence of good knowledge and positive attitudes towards monkeypox was 45.99% (95% CI: 36.98, 54.99) and 53.83% (95% CI: 42.96, 64.7), respectively. Significant heterogeneity was observed across studies; however, both statistical tests (Egger's test, p=0.1939; Begg's test, p=0.1982) indicated no significant publication bias excluded the presence of publication bias.

Conclusion: The knowledge and attitudes of healthcare workers towards monkeypox was low and unsatisfactory in sub-Saharan Africa. The review result underscores the need for targeted interventions to improve healthcare providers' understanding of mpox transmission, prevention, and management. Targeted educational programs and training are needed to improve the preparedness of healthcare workers.

Key Words: Attitude, healthcare workers, monkeypox, Mpox, Knowledge, Sub-Saharan Africa

Introduction

Mpox, a zoonotic disease endemic to Central and West Africa, poses a growing public health threat due to increased human-to-human transmission (1,2). Clinically, Mpox resembles smallpox and begins with symptoms like fever, headache, and muscle pain (1,3). Transmission to humans occurs through contact with infected ~~animal~~ fluids, skin lesions, or mucous membranes (2,4).

The recent global outbreaks prompted the WHO to declare Mpox a public health emergency of international concern in August 2024 (5), with Africa bearing a disproportionate burden(6,7), underscoring the global importance of coordinated prevention and control measures (1,5). As of June 2024, the WHO reported nearly 100,000 confirmed cases and over 200 deaths worldwide, with African nations disproportionately affected (6).

A solid foundation of knowledge, coupled with positive attitudes towards mpox, is a baseline for prevention and control of infection (8–10). Effective prevention and control of Mpox rely heavily on healthcare workers' (HCWs) knowledge and attitudes (11–13). However, studies showed suboptimal knowledge and attitudes among HCWs regarding Mpox, which may hinder effective management and control efforts (14–16).

Despite the critical role of HCWs, existing evidence suggests significant gaps in their knowledge and attitudes regarding Mpox, which need to be overcome through enhancing education and training (17,18). The situation in Sub-Saharan Africa, where the disease is endemic, requires careful examination due to the potential for unique challenges related to healthcare infrastructure (1,8,9,19,20). highlighting the urgent need for effective intervention strategies tailored to the specific contexts of the region (6).

The studies reported a significantly diverse proportion of mpox knowledge and attitudes of healthcare workers in Sub-Saharan countries (20–34). The inconsistent and varied findings across different regions highlight the critical need for a comprehensive understanding of the knowledge and attitudes of HCWs towards monkeypox in Sub-Saharan Africa. So, this systematic review and meta-analysis are essential to synthesize these diverse findings and provide robust evidence for developing targeted public health education and intervention programs.

Method

Protocol and Searching Strategy

The protocol was registered in PROSPERO (**ID: 1075654**), and the PRISMA guideline was followed to report the findings of the review (35). Seven databases, including PubMed/HINARI, were searched using MeSH terms. The full electronic databases were searched using: knowledge, attitude, "healthcare worker", "healthcare personnel", "healthcare providers", "monkey pox", mpox, and "sub-Saharan country" MeSH terms. The reference lists of all identified articles were checked for additional sources.

Eligibility criteria

All observational studies reporting knowledge, attitude, or both, and published in English up to June 25, 2025, were included in the meta-analysis. Conversely, studies conducted on non-HCW populations, qualitative studies, reviews, and case reports that we could not access in full text (despite two email requests to authors) and that were unrelated to the objective of our study were excluded from the review.

Outcome of Interest

This meta-analysis investigated knowledge and attitudes toward human Mpox among Sub-Saharan African HCPs, assessing pooled prevalence of "good" knowledge and "favorable" attitudes.

Good Knowledge: Adequate/good knowledge encompassing Mpox transmission, symptoms, treatment, prevention, and diagnosis (15).

Positive Attitudes: Defined as confidence in Mpox control, faith in preventive measures, and perception that actions were adequate to prevent spread (15).

Data extraction

A standardized Microsoft Excel spreadsheet prepared format was used to extract relevant data from the included primary studies. The following data were extracted by two reviewers (ML&YA) independently; first authors name, publication year, country, study design, sample size (total and included), response rate, number of HCW with good knowledge of mpox, proportion of good knowledge, proportion of positive attitudes, and number of HCW with positive attitudes towards mpox. Finally, reviewers independently verified the extracted data to ensure accuracy and eliminate incorrect information.

Study selection and quality Assessment

Two reviewers independently screened records using predefined criteria. Subsequently, the 3rd reviewer (AM) thoroughly reviewed the full texts of potentially eligible studies in the first review to determine if they met the inclusion criteria. Any disagreement between reviewers was solved by discussion, and if not, the third reviewer (AD) was engaged. The PRISMA flow diagram was used to summarize the study selection processes **(**As shown in Error! Reference source not found.**).** Quality was appraised with the (JBI) quality rating checklist, with studies scoring ≥6/9 included in the review **(**As shown in Error! Reference source not found.**)** (36).

Data analysis

The result of this meta-analysis was analyzed using STATA version 18. Heterogeneity across primary studies was checked using the standard I^2 statistical test. Random effect model was computed to estimate Der Simonian and Laird's pooled proportion of good knowledge and positive attitudes towards Mpox. Statistical tests (Egger's test and Begg's test) were used to assess publication bias. Furthermore, subgroup analysis was conducted based on country (Ethiopia and Nigeria), because these countries had sufficient published studies (N=6, E=5) for robust subgroup analysis, unlike other SSA nations with ≤2 studies. Forest plots were used to present the results of this meta-analysis.

Result

Study selection

Initially, 2,562 records were identified from various databases using the "MeSH" search combined with Boolean operators. Following the initial assessment, 2437 were excluded as irrelevant and unrelated to our study objectives, and 90 were excluded due to duplication and irrelevance. Further screening of 35 articles resulted in the exclusion of 18 articles, and after attempts to retrieve 17 articles, one remained unavailable. Sixteen articles underwent eligibility assessment; 1 article was

excluded from knowledge analysis, and 7 articles from attitude analysis because they did not report outcomes of interest. The final analyses included 15 articles (20,21,30–34,22–29) for knowledge and 8 articles (20,21,25,27–30,32) for attitude meta-analysis and review. The PRISMA flow diagram shows the study selection process (As shown in Error! Reference source not found.).

Characteristics of included studies

This meta-analysis and systematic review explored knowledge and attitudes about Mpox among healthcare providers in Sub-Saharan Africa, based on a total of 15 cross-sectional studies that assessed knowledge (n=5838, 91.8% response rate) and 8 cross-sectional studies on attitudes (n=3303, 94.9% response rate). Most of the included studies were conducted in Ethiopia and Nigeria, published since 2022, with sample sizes ranging from 164 HCWs (22) to 749 HCPs (28). The reported prevalence of good knowledge ranged from 22.5% (32) to 82.1% (30), while positive attitudes ranged from 35.8% (20) to 80% (25). **(**As shown in Error! Reference source not found.).

Quality of the included studies and publication bias

The qualities of independent studies related to knowledge and attitude were assessed using the JBI checklist for observational studies (36). It was determined that the studies are of moderate level or higher quality according to the JBI appraisal checklist **(**As shown in Error! Reference source not found.). The publication bias for knowledge articles was evaluated using Egger`s and Begg`s tests. Funnel plot inspection revealed slight asymmetry in the distribution of knowledge articles; however, both statistical tests (Egger's test, p=0.1939; Begg's test, p=0.1982) indicated no significant publication bias **(**As shown in **SF** Error! Reference source not found.**)**. Publication bias was not assessed for attitude articles due to the inclusion of fewer than ten studies, which is an insufficient number to evaluate through a test.

Pooled prevalence of Mpox knowledge among healthcare workers

The overall pooled prevalence estimate of good knowledge regarding Mpox among Sub-Saharan African HCWs was 45.99% [36.98, 54.99]. Significant heterogeneity (I^2 = 98.2%) was present, reflecting a wide range of variation in study settings. The overall effect test was statistically significant (z=10.01, ~~p<0.001~~) in spite of such heterogeneity. Individual point estimates ranged between 22.5% [17.07, 27.93](32) and 82.1% [78.35, 85.85] (As shown in Error! Reference source not found.**)** (30)**.**

Pooled prevalence of positive attitudes towards Mpox among healthcare workers

The individual study estimates of positive attitudes ranged from 35.8% (32.03, 39.57)(20) to 80% (75.74, 84.26) (25). The overall pooled estimate was 53.83% (42.96, 64.7), indicating that slightly more than half of HCWs have positive attitudes. High heterogeneity (I^2 = 97.72%) suggests that the findings are not consistent across the included studies **(**As shown in Error! Reference source not found.**)**.

Subgroup Analysis of Mpox knowledge by country

Country subgroup analysis compared HCWs' Mpox knowledge in Nigeria and Ethiopia. Ethiopian studies (n=5) reported prevalence rates from 28.13% (23.63, 32.63)(29) to 58.3% (54.42, 62.18) (20), with a pooled estimate of 46.07% (34.89, 57.25) (I^2 = 96.75%). Nigerian studies (n=6) showed more variation, ranging from 22.5% (17.07, 27.93) (32) to 82.1% (78.35, 85.85) (30), with an overall pooled prevalence of 47.55% (27.16, 67.93) and higher heterogeneity (I^2 = 99.03%) **(**As shown in Error! Reference source not found.**)**. This suggests similar levels of knowledge but potentially different underlying factors that influence them across the two settings.

Discussion

As monkeypox remains a public health emergency of international concern, especially in Africa (1,9), and numerous studies have explored healthcare providers' knowledge and attitudes, we conducted a meta-analysis to comprehensively assess the prevalence of good knowledge and positive attitudes, aiming to inform targeted awareness strategies.

This meta-analysis and systematic review revealed a pooled prevalence of good knowledge towards mpox among HCWs of 45.99% (95% CI: 36.98, 54.99). This finding relatively aligns with results from individual studies conducted in Cameroon (26) and Uganda (24),which reported prevalence rates of 42.1% and 44%, respectively. However, our meta-analysis demonstrated a significantly higher pooled prevalence of adequate knowledge compared to previous systematic reviews and meta-analyses that reported 26% (14) and 32% (15). This improved knowledge may be attributable to increased awareness, heightened media coverage, targeted educational initiatives, and greater access to information for HCWs following the recent global outbreaks of mpox in 2022. Crucially, improving knowledge about mpox necessitates identifying reliable and up-to-date information sources that provide accurate data regarding transmission, symptoms, prevention, and treatment (37).

Moreover, our pooled estimate of good knowledge among HCWs was significantly higher than the prevalence rates reported in studies conducted in Nigeria (32), Cameroon (26), and Ethiopia (21), which found prevalence rates of 22.5%, 42.1%, and 38.5%, respectively. The most probable reason for this improved knowledge is that our study included research conducted after the 2022 global outbreaks, whereas the previous meta-analyses incorporated studies carried out before these events, when awareness and readily available information were more limited.

This meta-analysis estimate showed the lower pooled prevalence of good knowledge about human mpox among healthcare workers (45.99%) compared to several individual studies conducted in

Nepal (38), Vietnam(18), Saudi Arabia (39), Egypt(40), Uganda (25), Nigeria (30), and Ethiopia (28), which reported [illegible]4%, 50%, 55%, 55.5%, 60%, 82.1%, and 56.5% prevalence respectively. The discrepancy could be attributed to the fact that a meta-analysis combined and pooled data from multiple individual studies; as a result, the lower pooled estimate suggests that the influence of these studies with higher prevalence might have been counterbalanced by other studies included in the meta-analysis with lower reported knowledge levels. Another possible explanation for the lowered pooled estimate of the meta-analysis might be due to individual studies focused on particularly informed or motivated healthcare providers, unlike the broader Sub-Saharan context.

The pooled prevalence of positive attitudes towards mpox viral infection among healthcare workers was 53.83% (95%: 42.96, 64.7). The finding of a pooled estimate of positive attitude is approximately consistent with a study conducted in Nepal (38), which reported 51.1% ~~of positive attitude towards monkeypox,~~ and is comparable to a meta-analysis study with 53% ~~(15)~~ pooled prevalence ~~of positive attitudes among healthcare workers~~.

The ~~pooled~~ estimate of positive attitudes of HCWs towards ~~human~~ monkeypox in this meta-analysis is ~~a~~ higher ~~prevalence~~ compared to the meta-analysis and systematic review, with 34.6% ~~prevalence of positive attitudes~~ (14). The potential reason for the discrepancy could be due to ~~previous meta-analyses and systematic reviews conducted before a major surge in awareness and concern about human monkeypox viral infection, potentially encompassing a period when the perceived threat was lower.~~

[illegible]s meta-analysis's pooled estimate of meta-analysis is also significantly higher prevalence of positive attitudes towards monkeypox among HCWs compared to individuals conducted in Vietnam (18), Egypt (40), Nigeria (32), and Ethiopia (27), which reported 33%, 44.5%, 44.5% and 49.2% prevalence of positive attitudes, respectively. The potential difference might be because

attitudes are shaped by cultural values, belief and social norms and the individual studies may reflect specific cultural factors that influence attitudes towards infectious disease among healthcare workers (HCWs.) Furthermore, the pooled estimates reflects an overall pooled attitudes which could be influenced by the inclusion of studies with extremely more positive attitude scores, thereby diluting the impact of those with lower prevalence.

However, our meta-analysis pooled estimate prevalence of positive attitudes towards mpox among healthcare workers (HCWs) is lower compared to studies conducted in Nigeria (30), Uganda (25) and Ethiopia (21), which reported [illegible]9%, 80%, and 62% prevalence of positive attitudes towards mpox among healthcare workers, respectively. The meta-analysis pools data from a broader range of studies, potentially including those with less positive attitude scores that ~~dilute~~ the pooled estimate. Moreover, the individual studies might have been conducted in specific settings or among healthcare providers with higher perceived risk, specialized training, or a stronger sense of preparedness, which could result in more positive attitudes than the pooled estimate prevalence of positive attitudes towards human monkeypox across Sub-Saharan Africa.

Limitations of the study

This systematic review and meta-analysis, while providing valuable insights into healthcare provider knowledge and attitudes towards mpox in Sub-Saharan Africa, has limitations. Restricting inclusion to English-language articles may introduce language bias. The cross-sectional study designs could be affected by confounding variables. Rapidly evolving knowledge about mpox means that included studies might not fully reflect the most up-to-date information, potentially introducing heterogeneity, even after using subgroup analysis and random effects modeling. Variations in questionnaire design, distribution, and participant demographics also present challenges. Moreover, variability in cultural, socioeconomic, and governmental factors

may influence the outcomes. Due to limited data, subgroup analyses were restricted, hindering country-specific conclusions. The small number of attitude studies precluded subgroup analysis and publication bias assessment, limiting robustness. These constraints warrant cautious interpretation and highlight the need for further research. However, robust quality assessment tools (JBI), adherence to PRISMA guidelines, Egger's test, and Begg`s test were employed; furthermore, independent data extraction and article selection were performed, strengthening the validity of the results.

Conclusion

The knowledge and attitudes towards Mpox among healthcare providers in Sub-Saharan Africa were low and unsatisfactory. Specifically, this meta-analysis reveals a low pooled estimate of good knowledge towards Mpox, and relatively improved attitudes among Sub-Saharan African healthcare workers regarding Mpox, underscoring the need for targeted interventions. The relatively low knowledge of healthcare workers necessitates improved understanding of mpox transmission, prevention, and management, tailored to address specific gaps and cultural contexts. These efforts are crucial for strengthening Mpox control and public health preparedness in the region.

Abbreviations

HCWs: Healthcare workers, **JBI:** Joanna Briggs Institute, **Mpox:** Monkeypox **PRISMA:** Preferred reporting items for systematic reviews and meta-analysis**, WHO:** World Health Organization

Declarations

Ethics approval and consent to participate

Not applicable.

Consent for publication

Not applicable.

Availability of data

Data will be provided by the corresponding author upon reasonable request.

Competing interests

The authors have declared that there are no competing interests.

Funding

No funding was obtained for this study.

Author contributions

ML: Conceptualization, data curation, formal analysis, project administration, methodology, writing original draft, writing-review & editing.

AW& AD: data curation, resources and software

YA: data curation, methodology and resources

HA, AY &AM: Supervision and validation

All authors read and approved the final draft of the manuscript.

Acknowledgment

Not applicable.

References

1. African Union; Centres for disease control and prevention. [illegible]NKEYPOX TECHNICAL FACTSHEET. Africa CDC. 2022;1–8.

2. Jokar M, Rahmanian V JK. Monkeypox outbreak reflecting rising search trend and concern in nonendemic countries: a Google trend analysis. Disaster. Med Public Heal Prep. 2023;17:286.

3. Human monkeypox. Human monkeypox: an emerging zoonosis - The Lancet Infectious Diseases. https:// www. thela ncet. com/ journ als/ laninf/ artic le/ PIIS1 473- 3099(03) 00856-9/ fullt ext. Lancet Infect Dis.

4. Sabeena S. The changing epidemiology of monkeypox and preventive measures: an update. Arch Virol. 2023;168(1):31.

5. First Meeting of the International Health Regulations (2005) Emergency Committee. Regarding the Upsurge of mpox 2024. 2024; Available from: https://www.who.int/news/item/19-08-2024-first- meeting-of-the-international-health-regulations-(2005)-emergency- committee-regarding-the-upsurge-of-mpox-2024. 2024;

6. Multi-Country Outbreak of Mpox ESRM 2024. https://www.who.int/publications/m/item/multi- country-outbreak-of-mpox–external-situation-report-33--31. https://www.who.int/publications/m/item/multi- country-outbreak-of-mpox–external-situation-report-33--31. 2024;

7. Akingbola A, Adegbesan CA, Adewole O, Idahor C, Odukoya T, Nwaeze E, et al. Understanding the resurgence of mpox : key drivers and lessons from recent outbreaks in Africa. Trop Med Health [Internet]. 2025;1:1–12. Available from: https://doi.org/10.1186/s41182-024-00678-1

8. World Health Organization (WHO). Vaccines and immunization for monkeypox: interim guidance, 14 June 2022. World Heal Organ Geneva. 2022;

9. World Health Organization (WHO). Mpox Multi-country external situation report no.53 published 29 may 2025. World Heal Rep. 2025;(53):1–30.

10. Bass J, Tack DM, McCollum AM et al. Enhancing health care worker ability to detect and care for patients with monkeypox in the Democratic Republic of the Congo. Int Heal. 2013;5(4):237–43.

11. Sklenovská N VRM. Emergence of monkeypox as the most important orthopoxvirus infection in humans. Front Public Heal. 2018;6:241.

12. Pope JP, Pelletier L GC. Starting off on the best foot: a review of message framing and message tailoring, and recommendations for the comprehensive messaging strategy for sustained behavior change. Heal Commun. 2018;33(9):1068–77.

13. Jahromi AS, Jokar M, Sharifi N et al. Global knowledge, attitude, and practice towards COVID-19 among pregnant women: a systematic review and meta-analysis. BMC

Pregnancy Childbirth. 2023;23(1):278.

14. Sotoodeh JA, Mohammad J, Nader S, Sirus K, Karamatollah R, Vahid R. Global knowledge and attitudes towards mpox (monkeypox) among healthcare workers : a systematic review and meta-analysis. Int Health [Internet]. 2024;16(October 2023):487–98. Available from: https://doi.org/10.1093/inthealth/ihad094

15. Barboza JJ, Siddiq A, Sah R, Leo DA, Id MJV, Id AJR. Knowledge and attitude towards mpox : Systematic review and meta-analysis. PLoS One. 2024;19(8):1–15.

16. Harapan H, Setiawan AM, Yufika A et al. Knowledge of human mon- keypox viral infection among general practitioners: a cross-sectional study in Indonesia. Pathog Glob Heal. 2020;114(2):68–75.

17. Sahin TK, Erul E, Aksun MS, Sonmezer MC, Unal S. Knowledge and Attitudes of Turkish Physicians towards Human Monkeypox Disease and Related Vaccination : A Cross-Sectional Study. Vaccines. 2023;1–16.

18. Thi H, Nguyen N, Nguyen T Van, Tran K Van. Healthcare Providers ' Knowledge , Attitude , and Practices Regarding Prevention of Mpox in Vietnam. SAGE Open. 2024;(December):1–11.

19. Lounis M, Hamimes A. Assessment of Monkeypox (MPOX) Knowledge and Vaccination Intention among Health and Life Sciences Students in Algeria : A Cross-Sectional Study. Infect Dis Rep. 2024;170–80.

20. Yeshiwas AG, Temesegen A, Melkie G, Tsega TD, Mola A, Tesfa H, et al. Assessing healthcare workers ' confidence level in diagnosing and managing emerging infectious virus of human mpox in hospitals in Amhara Region , Northwest Ethiopia : multicentre sectional study. 2024;

21. Aynalem ZB, Abate MD, Meseret F, Muhamed N, Abebe GK, Adal AB, et al. Knowledge , Attitude and Associated Factors of Monkeypox Infection Among Healthcare Workers in Injibara General Hospital , Northwest Ethiopia Knowledge , Attitude and Associated Factors of Monkeypox Infection Among Healthcare Workers in Injibara General H. J Multidiscip Healthc. 2025;2390.

22. Iwuafor AA, Owai PA, Udoh AU, Ogban GI, Ita IO EE. Assessing Medical Doctors ' Knowledge and their Confidence in Spot Diagnosing Monkeypox in. Covenant J Heal LIFE Sci. 2023;1(1):29–41.

23. Epipode Ntawuyamara, Thierry Ingabire, Innocent Yandemye, Polycarpe Ndayikeza, Bina Bhandari Y-HL. Assessing healthcare workers' knowledge and confidence in the diagnosis, management and prevention of Monkeypox. World J Clin Cases. 2025;13(1):1–11.

24. Namakula NO, Migisha R, Kwesiga B AA. Mpox knowledge , vaccine attitudes , and vaccine concerns among healthcare workers , Kampala Metropolitan Area ,. Uganda Public Heal Bull. 2025;10(2).

25. Joyce A, Kobusingye O, Mfitundinda E, Katumba H, Nalwanga J, Wenani D, et al. Knowledge attitude and practices toward mpox among healthcare workers in Kasese and Mayuge districts , Uganda ,. Uganda Public Heal Bull. 2025;10(1):1–10.

26. Nka AD, Bouba Y, Fokam J, Christelle A, Gabisa JE, Mandeng N, et al. Current knowledge of human Mpox viral infection among healthcare workers in Cameroon calls for capacity-strengthening for pandemic preparedness. Front Public Heal. 2024;(12):1288139.

27. Beyna AT, Yefter ET, Asrie AB, Ayele HS, Belete TM, Ayenew W, et al. Assessment of healthcare workers knowledge and attitudes towards Mpox infection at University of Gondar Comprehensive Specialized Referral Hospital ,. Front Public Heal. 2025;10(March).

28. Fetensa G, Wakuma B, Besho M, Yadesa G, Gugsa J, Tufa DG, et al. Improving control of the Mpox outbreak : a national cross-sectional study on the knowledge , attitudes , and influencing factors among frontline healthcare professionals in Ethiopia. Front Public Heal. 2025;(July):1–10.

29. Kiros T etal. Assessment of Knowledge , Attitude , and Associated Factors of Mpox Among Healthcare Professionals at Debre Tabor Comprehensive Specialized Hospital , Northwest Ethiopia , 2024 : A Cross - Sectional Study. Heal Sci Reports Orig. 2025;1–15.

30. Abdulmumin AK, Adeniyi MA, Temitayo-oboh AO, Abdullah A, Ojo OY. Knowledge , perception and safety practices of monkeypox infection among healthcare workers in a

tertiary health facility in southwest Nigeria Knowledge , perception and safety practices of monkeypox infection among healthcare workers in a tertiary health. Int J Community Med Public Heal. 2023;(August).

31. Oche OM, Abdulaziz MD, Wali A, Muhammad H YH. Knowledge and preventive practices related to Monkey pox among medical doctors in Sokoto metropolis of Sokoto State, Nigeria. IBOM Med J. 2024;17(1):15–24.

32. Orok E, Adele G, Oni O, Adelusi A, Bamitale T, Jaiyesimi B, et al. Assessment of knowledge and attitude of healthcare professionals towards Mpox in a Nigerian hospital. Sci Reports |. 2024;1–14.

33. Ajayi PO, Esan DT, Ipinnimo TM, Olanrewaju MT, Solomon OO, Atanda-owoeye OO. Knowledge and awareness of human mpox infection among healthcare workers : A cross-sectional study in southwestern Nigeria. Asian Pac J Trop Med. 2023;16(December 2022):245–52.

34. Uche Eze E. Assessment of the knowledge of healthcare workers on monkeypox in Nigeria Bewertung der Kenntnisse des Gesundheitspersonals über Affenpocken. GMS Hyg Infect Control 2024,. 2024;19:1–15.

35. Liberati A, Altman DG, Tetzlaff J, Mulrow C, Gøtzsche PC, Ioannidis JP et al. The PRISMA statement for reporting systematic reviews and meta-analyses of studies that evaluate health care interventions: explanation and elaboration. J Clin Epidemiol. 2009;62(10):34.

36. Appraisal C, Reviews JBIS. CHECKLIST FOR Critical Appraisal tools for use in JBI Systematic Reviews. JBI.global. 2020;

37. Leo´n-Figueroa DA, Barboza JJ V-GM. Sources of information on monkeypox virus infection. A systematic review with meta-analysis. BMC Public Health. 2024;24:276.

38. Rodriguez-morales AJ, Barboza JJ. Socio-demographic determinants of the knowledge and attitude of Nepalese healthcare workers toward human monkeypox : a cross-sectional study. Front Public Heal. 2023;(May):1–8.

39. Alshahrani NZ, Algethami MR, Alarifi AM, Alzahrani F, Alshehri EA, Alshehri AM, et al.

Knowledge and Attitude Regarding Monkeypox Virus among Physicians in Saudi Arabia : A Cross-Sectional Study. Vaccines. 2022;1–13.

40. Amer FA, Nofal HA, Gebriel MG, Bedawy AM, Allam AA, Khalil HES, et al. Grasping knowledge , attitude , and perception towards monkeypox among healthcare workers and medical students : an Egyptian cross- sectional study. Front Cell Infect Microbiol. 2024;(14):1–10.

Figure
Click here to access/download;Figure;Figure 1 PRISMA flow diagram.docx

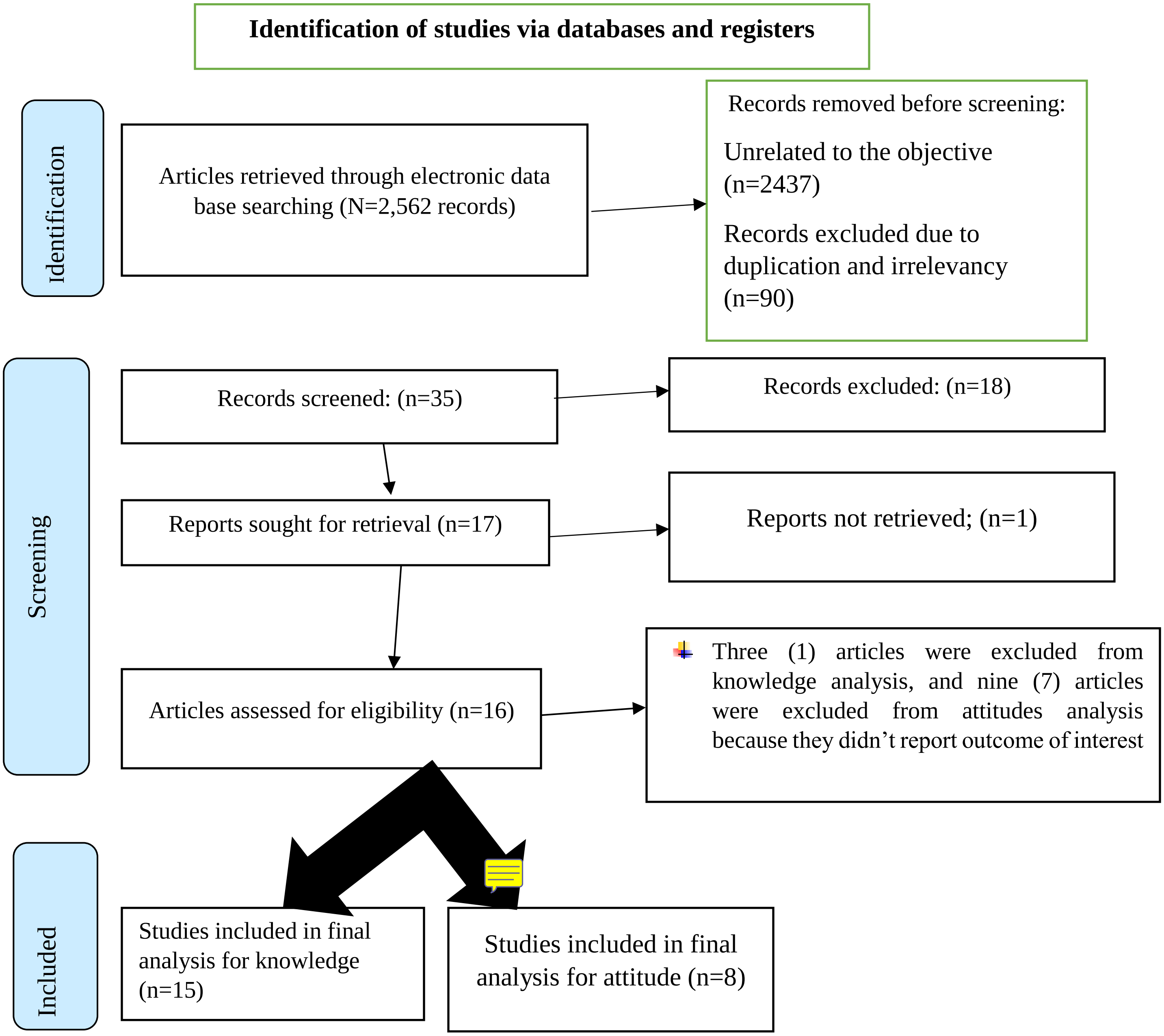

Figure 1: PRISMA flow diagram of included studies in the analysis to estimate the pooled prevalence of good knowledge and positive attitudes towards mpox in Sub-Saharan Africa

Figure
Click here to access/download;Figure;Figure 2 Forest plot of pooled good knowledge.docx

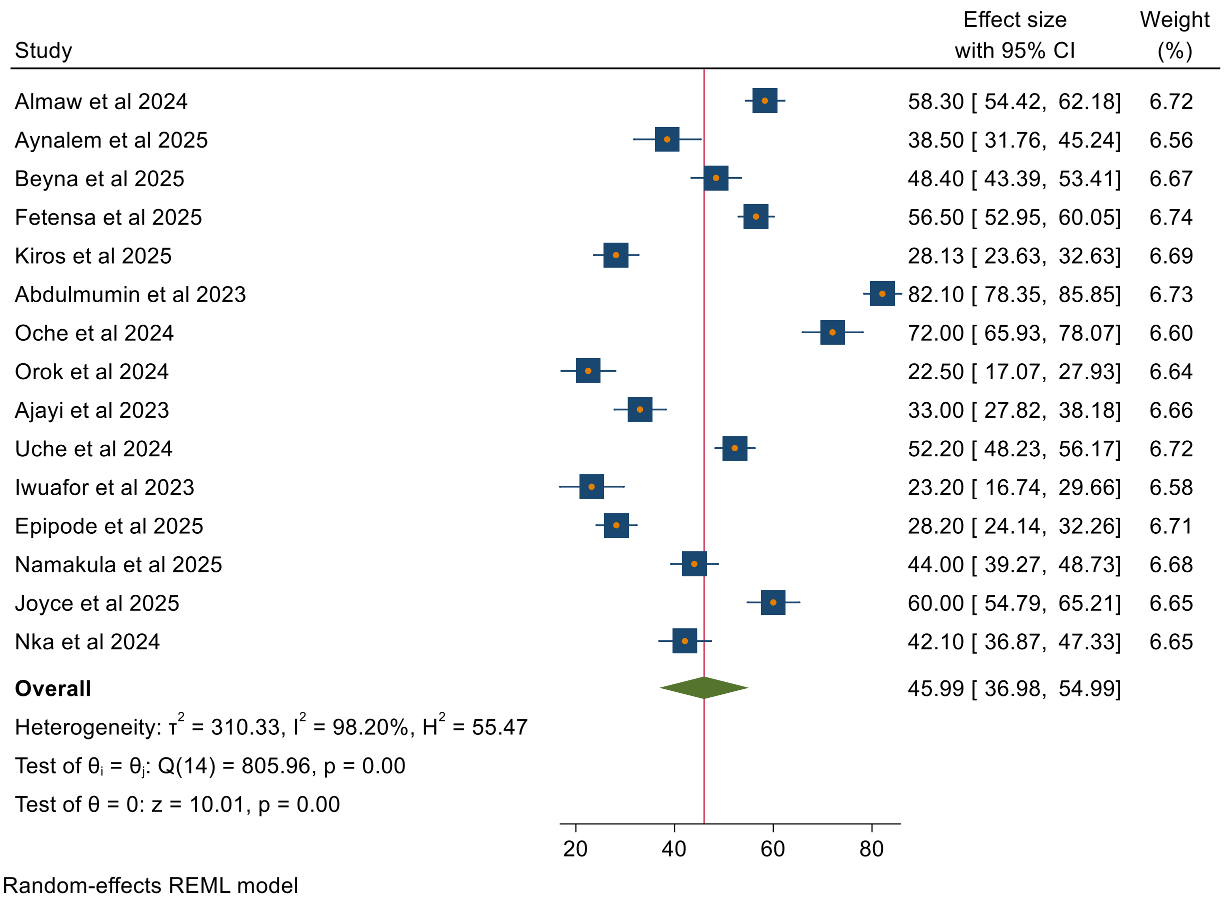

Figure 1: Forest plot of pooled prevalence of good knowledge towards Mpox among healthcare workers (HCW) in Sub-Saharan Africa

Figure
Click here to access/download;Figure;Figure 3 Forest plot of the pooled attitude.docx

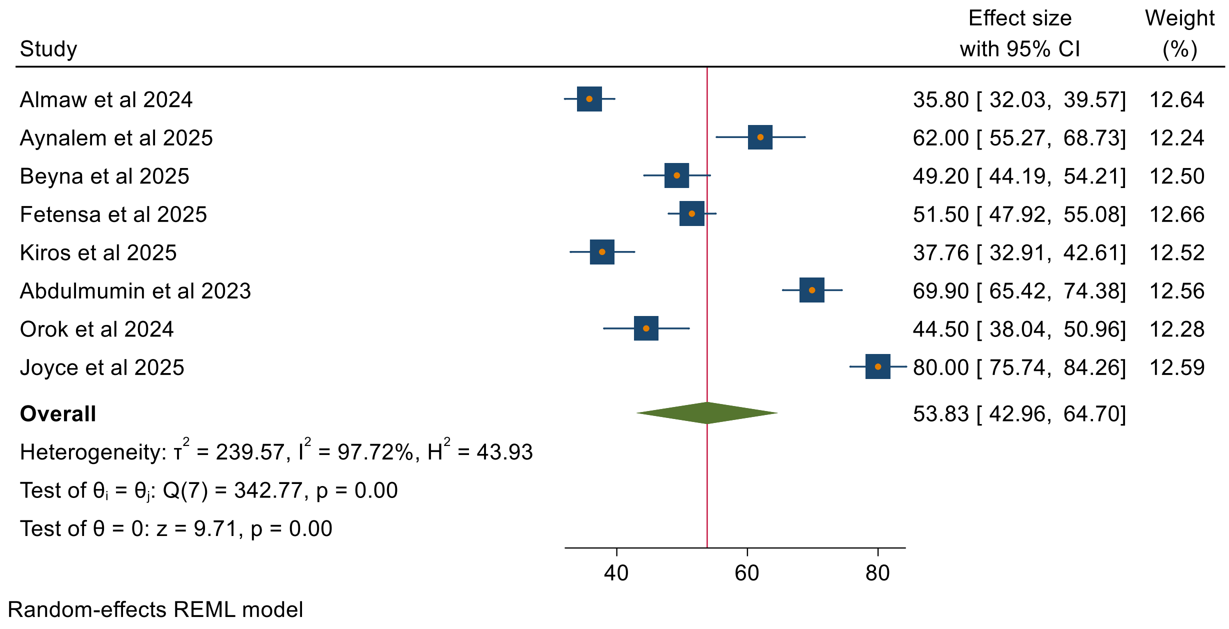

Figure 1: Forest plot of the pooled prevalence of positive attitude towards Mpox among healthcare workers in Sub-Saharan Africa.

Figure
Click here to access/download;Figure;Figure 4 Forest plot Subgroup analyses.docx

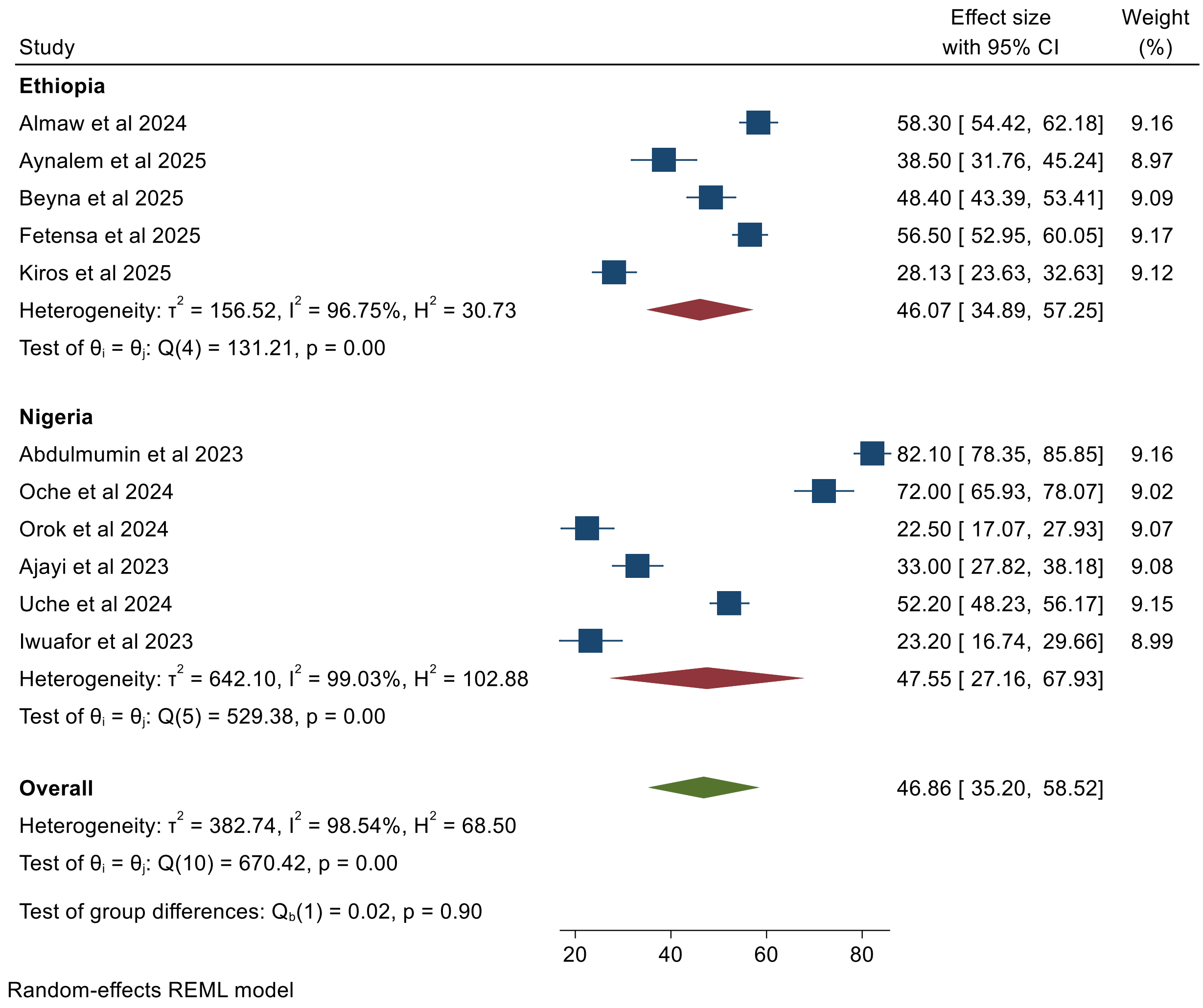

Figure 1: Forest plot comparing the pooled prevalence of good knowledge towards Mpox among healthcare workers in Ethiopia and Nigeria.

Figure

Click here to access/download;Figure;Figure 5 SF Funnel plot for publication bias.docx

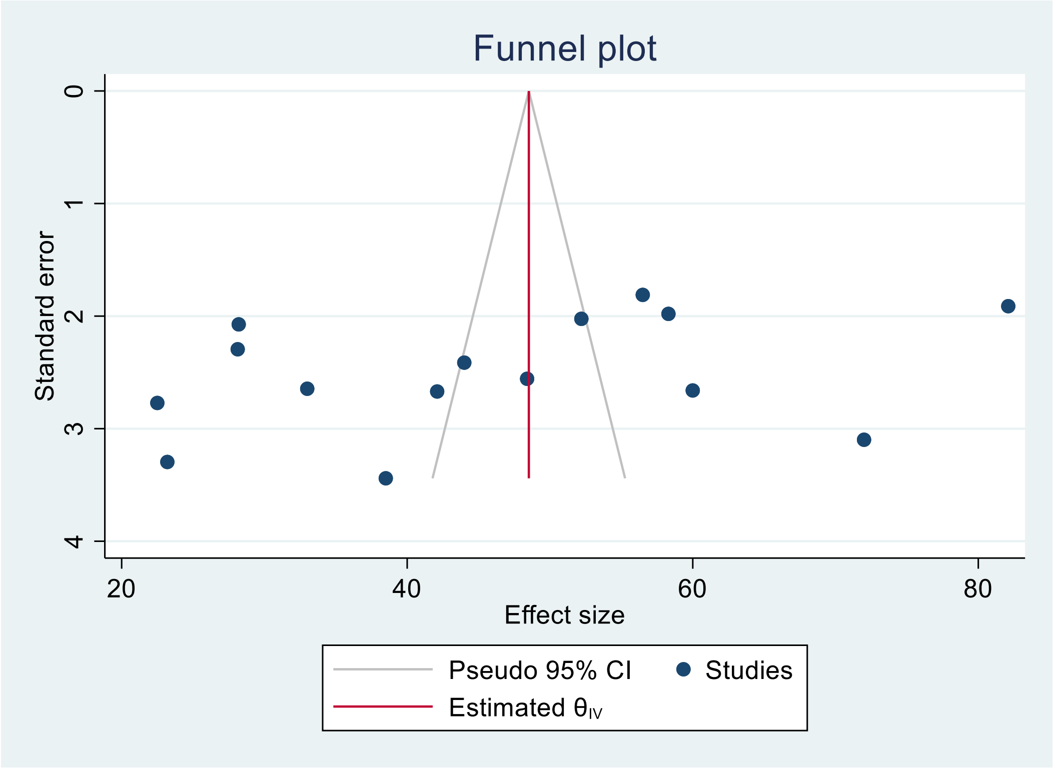

Figure 1: Funnel plot assessing potential publication bias in the meta-analysis of healthcare workers (HCW) knowledge towards Mpox in Sub-Saharan Africa.

Table

Click here to access/download;Table;Table 1 Characteristics and JBI score.docx

Table 1: Characteristics and quality (JBI score) of the included studies on knowledge and attitude analysis

Author	Year	Country	Study design	Total sample	Response rate	Included sample	Number of study participants with good knowledge	Prevalence of good knowledge	Number of participants with positive attitude	Prevalence of +ve attitude	JBI score
Almaw et al(20)	2024	Ethiopia	Cross section	640	96.9%	620	361	58.3	222	35.8%	6
Aynalem et al(21)	2025	Ethiopia	Cross sectional	201	99.5%	200	77	38.5%	124	62%	7
Beyna et al(27)	2025	Ethiopia	Cross sectional	401	95.3%	382	185	48.4%	188	49.2%	7
Fetensa et al(28)	2025	Ethiopia	Cross sectional	800	94%	749	423	56.5%	386	51.5%	6
Kiros et al(29)	2025	Ethiopia	Cross sectional	384	100%	384	108	28.13%	145	37.76%	8
Abdulmumin et al(30)	2023	Nigeria	Cross sectional	402	100%	402	330	82.1%	281	69.9%	6
Oche et al(31)	2024	Nigeria	Cross sectional	210	100%	210	151	72%	NR	NR	6
Orok et al(32)	2024	Nigeria	Cross sectional	227	100%	227	51	22.5%	101	44.5%	6
Ajayi et al(33)	2023	Nigeria	Cross sectional	338	93%	316	107	33%	NR	NR	6
Uche et al(34)	2024	Nigeria	Cross sectional	609	100%	609	318	52.2%	NR	NR	7
Iwuafor et al(22)	2023	Nigeria	Cross sectional	450	36.4%	164	38	23.2%	NR	NR	7
Epipode et al(23)	2025	Burundi	Cross sectional	471	100%	471	330	70%	NR	NR	7
Namakula et al(24)	2025	Uganda	Cross sectional	423	100%	423	186	44%	NR	NR	6
Joyce et al(25)	2025	Uganda	Cross sectional	426	80%	339	202	60%	270	80%	8
Nka et al(26)	2024	Cameroon	Cross sectional	377	90.7%	342	144	42.1%	NR	NR	7

NR: No outcome of interest (attitude) reported

Click here to access/download
Supporting Information
Prisma check list 2025.doc
