# Supplementary material for: Mpox knowledge and positive attitudes in Sub-Saharan African healthcare workers after 2022 outbreak of disease: A systematic review and meta-analysis
Source: PLoS Negl Trop Dis. 2026 Feb 9;20(2):e0013977. doi: 10.1371/journal.pntd.0013977 (PMC12900440; doi:10.1371/journal.pntd.0013977)
Supplement: Attachment — Submitted filename: Response to Reviewers.pdf [file pntd.0013977.s007.pdf]

RESPONSE TO REVIEWERS

Manuscript ID: PNTD-D-25-01469

Title: Mpox Knowledge and Positive Attitudes in Sub-Saharan African Healthcare Workers After 2022 Outbreak of Disease: A Systematic Review and Meta-Analysis.

Journal: PLOS Neglected Tropical Diseases

Dear Editors and Reviewers,

Thank you for the opportunity to revise and resubmit our manuscript. We sincerely appreciate the thoughtful and constructive feedback provided by the editors and reviewers, which has significantly strengthened our work. We have carefully addressed each comment and have incorporated all suggested revisions. Below, we provide a point-by-point response to each comment, detailing the changes made in the manuscript. All line and page numbers refer to the revised manuscript file.

Methods

Reviewer 1

Major comments

Reviewer comment 1: Objectives and Hypothesis

The objective is stated clearly, but the manuscript does not present a formal testable hypothesis. Please reframe your objective into a hypothesis.

Author`s response: Thank you for your valuable feedback regarding the need for a formal testable hypothesis. In response, we have reframed the objective into the following explicit hypothesis:

Hypothesis: We hypothesize that the pooled prevalence of good knowledge and positive attitudes toward mpox among healthcare workers in Sub-Saharan Africa after the 2022 outbreak is below 50%.

Objective: This systematic review and meta-analysis was aimed to determine the pooled prevalence of good knowledge and positive attitudes toward mpox among healthcare workers in Sub-Saharan Africa following the 2022 outbreak.

These revisions have been incorporated into the manuscript on page 5, lines 90–96. We believe this adjustment strengthens the study's conceptual clarity and aligns with standard research reporting practices. Thank you again for your constructive input.

Reviewer comment 2: Heterogeneity in Results
The pooled estimates show very high heterogeneity (over 95%). This means the studies differ too much to be easily combined. Conduct additional analyses such as subgroup analysis or meta-regression (by year, study quality, or type of healthcare worker).

Author`s response: Thank you for your insightful observation regarding the high heterogeneity across included studies. We have conducted the following additional analyses to explore potential sources of heterogeneity:

- **Meta-regression** was performed using publication year and sample size as continuous moderators. Results indicated that neither variable significantly explained the between-study variance (R^2 = 0.63% for publication year and 2.07% for sample size), suggesting that temporal trends and study scale do not account for the observed heterogeneity (page-13, line 231-236).
- **Sensitivity analysis** confirmed that no single study exerted undue influence on the pooled prevalence estimate for good knowledge (page-13, line 238-240).

Despite these analyses, substantial heterogeneity remains, likely attributable to unmeasured methodological and contextual factors such as variations in assessment tools, healthcare infrastructure, regional training exposure, and study setting. We have explicitly acknowledged this limitation in the manuscript and advice that the pooled estimates be interpreted with appropriate caution (page-16 & 17, lines 299-318). We appreciate your guidance in strengthening the analytical rigor of this review.

Reviewer comment 3: Study Selection and Figures
Several parts of the manuscript show "Error! Reference source not found" instead of actual figures or tables. This makes it difficult to follow the selection process and the results.
Please correct and insert all missing figures (e.g., PRISMA flow diagram, forest plots).

Author`s response: Thank you for bringing this formatting issue to our attention. We apologize for the placeholder errors in the previous version of the manuscript. All figures and tables, including the PRISMA flow diagram and forest plots, have now been correctly uploaded as figure, captioned and properly cited in the manuscript text. This ensures that the study selection process and results are clearly presented and easy to follow. We appreciate your diligence, which has significantly improved the readability of our work.

Reviewer comment 4: Ethical Considerations
As a review, new ethics approval was not required, which is correct.
However, it is not clear whether all included studies reported ethical approval and participant consent. State whether all studies reported ethical clearance. If not, please mention this as a limitation.

Author`s response: Thank you for this important clarification point. We confirm that while no new ethical approval was required for this systematic review and meta-analysis, majority of included primary studies reported obtaining appropriate ethical clearance from their respective institutional or national review boards prior to data collection, but one primary study was not clearly reported ethical statements (Iwuafor, 2023). This acknowledged as a limitation. This assurance has been added to the manuscript in the Declarations section (page 18, lines 344–348). We appreciate your guidance in ensuring the ethical transparency of our work.

Minor comments

Reviewer comment 5: Some references are incomplete, repeated, or not in correct Vancouver style. Please revise and standardize them. The manuscript has a few grammatical and tense errors. These should be corrected during editing. The Discussion section repeats some of the Results and should be shortened.

Author`s response: Thank you for your thorough review and constructive feedback. We have addressed each point as follows:

- **References**: All references have been revised and standardized in full Vancouver style, with duplicates removed and incomplete entries corrected.

- **Language and grammar**: The manuscript has been carefully edited to correct grammatical and tense errors throughout.
- **Discussion section**: The Discussion has been revised to reduce repetition of results and to present a clearer, more concise synthesis of findings and their implications.

We believe these revisions have significantly strengthened the clarity and academic quality of the manuscript.

Reviewer 2

Reviewer comment 1: Clear registration (PROSPERO), PRISMA compliance, multiple databases, inclusion/exclusion criteria, JBI tool for appraisal, random-effects model, subgroup analysis (Ethiopia vs. Nigeria).
However, seven databases were mentioned, but strategies were not reported clearly. Full reproducible search strings should be included. Restriction to English-only is a limitation that introduces potential bias and should be acknowledged more explicitly. "JBI checklist" is mentioned repeatedly across sections, which is redundant and disrupts flow. It should be described once in Methods, summarized in Results, and the abbreviation placed properly in the Abbreviations section. Subgroup analysis was limited to two countries; additional sensitivity analyses (e.g., by year, sample size, study quality) could strengthen reliability.

Author`s response: Thank you for your comprehensive and constructive feedback. We have addressed your suggestions as follows:

- **Search Strategy**: Full, reproducible search strings for all seven databases have been included as **Supplementary File 2**.

- **Language Restriction**: The potential bias introduced by restricting the review to English-language publications has been explicitly acknowledged as a limitation.

- **JBI Checklist Reporting**: The use of the Joanna Briggs Institute (JBI) tool is now described once in the Methods, summarized in Results, and the abbreviation appears in the Abbreviations section to improve flow and avoid redundancy.

- **Additional Analyses**: Meta-regression was conducted using publication year and sample size as moderators. Although these variables did not significantly explain the observed heterogeneity, the analysis is reported transparently. Sensitivity analysis confirmed that no single study unduly influenced the pooled estimate for knowledge.

We believe these revisions strengthen the methodological clarity, transparency, and robustness of our systematic review. Thank you for your valuable guidance.

Results

Reviewer 1

Reviewer comment 1: Study Selection

(Line 121–126) The authors explain how many studies were found and excluded. This is good. But the PRISMA diagram is missing and shows "Error! Reference source not found." This makes it hard to follow. Please add the PRISMA diagram.

Author`s response: Thank you for pointing out the missing PRISMA diagram. We apologize for the placeholder error in the previous version. The PRISMA diagram (Fig 1) has now been inserted into the manuscript with a complete caption and is properly referenced in the text on page 10, line 185-186. This addition ensures full transparency and clarity in reporting the study selection process. We appreciate your guidance in improving the manuscript's presentation and readability.

Reviewer comment 2: Characteristics of studies
(Line 132–137) The description of studies (sample size, response rate, country) is useful. But it is long and heavy to read. Most of this information should be in Table 1 with clear formatting.
(Line 137–139) The ranges for knowledge (22.5%–82.1%) and attitudes (35.8%–80%) are given. But the forest plot is missing and replaced with "Error! Reference source not found." Please insert the forest plot.

Author`s response: Thank you for your valuable feedback on improving the presentation of study characteristics and results. We have implemented the following revisions:

- **Study characteristics** have been summarized concisely in the text (page 10, lines 188–192), with full details presented in a clearly formatted Table 2.
- **Forest plots** for both knowledge (Fig 3) and attitudes (Fig 4) have been inserted into the manuscript and properly referenced (page 12, lines 211–212 for knowledge; page 12, lines 217–218 for attitudes). Placeholder errors have been corrected.

These adjustments enhance readability and provide clear visual representation of the pooled estimates and their variability. We sincerely appreciate your suggestions, which have strengthened the clarity and presentation of our findings.

Reviewer comment 3: Study Quality and Publication Bias
(Line 140–143) The quality of studies is mentioned, but again a figure is missing. Please add the table or figure showing quality scores.
(Line 144–148) The results of Egger's and Begg's tests are clear (no publication bias). But the funnel plot is missing. Please add the funnel plot to support this result.

Author`s response: Thank you for your constructive feedback on study quality and publication bias reporting. We have addressed your comments as follows:

- **Quality assessment** is now presented in a clearly formatted Table 1 (page 8), which includes the detailed Joanna Briggs Institute (JBI) checklist results for all included studies.
- **Funnel plot** (Fig 2) has been inserted into the manuscript with a complete caption and is properly referenced to visually support the Egger's and Begg's test results.

These additions ensure full transparency in reporting methodological quality and publication bias assessment. We appreciate your guidance in enhancing the completeness and clarity of our manuscript.

Reviewer comment 4: Pooled Prevalence of Knowledge
(Line 150–153) The pooled knowledge prevalence (45.99%) is reported clearly with CI and I^2 value. This is good.
(Line 154–156) The knowledge range (22.5%–82.1%) is stated. But the figure is missing again. Please add the forest plot.

Author`s response: Thank you for highlighting the missing forest plot for the knowledge analysis. We have now uploaded the forest plot (Fig 3) into the manuscript with a complete caption and proper citation (page 12). This figure visually represents the pooled prevalence estimate and study-specific contributions. We apologize for the earlier omission and appreciate your careful review, which has improved the completeness of our results presentation.

Reviewer comment 5: Pooled Prevalence of Attitudes (Lines 157–162)
9. (Line 157–159) The pooled attitude prevalence (53.83%) is clear. Good reporting.
10. (Line 160–162) High heterogeneity ($I^2 = 97.72\%$) is reported. This is important. But the forest plot is missing. Please insert the forest plot.

Author`s response: Thank you for noting the missing forest plot for the attitude analysis. We have now uploaded the forest plot (Fig 4) into the manuscript with a complete caption and proper citation (page 12). This figure visually presents the pooled prevalence of positive attitudes and the corresponding heterogeneity across studies. We apologize for the earlier oversight and appreciate your valuable input, which has enhanced the clarity and transparency of our results.

Reviewer 2

Reviewer comment 1: Results structured (study selection, characteristics, quality, pooled prevalence, and subgroup analysis). Use of forest and funnel plots is appropriate.
Several "Error! Reference source not found" placeholders reduce readability. Figures and tables need better resolution and consistent labeling. Percentages and CIs are inconsistently reported (mixed decimals), and p-values vary in precision. Recommend standardizing: percentages and CIs to one decimal, p-values to two or three decimals consistently.

Author`s response: Thank you for your detailed and constructive feedback. We have addressed each point as follows:

- **Placeholder errors**: All "Error! Reference source not found" placeholders have been replaced with correctly inserted and referenced figures and tables.
- **Figure and table quality**: All visuals have been updated with improved resolution and consistent labeling throughout the manuscript.

- **Numerical standardization**: Percentages and confidence intervals are now reported to one decimal place, and p-values to two or three decimals, ensuring consistency and precision in reporting.

We believe these revisions significantly enhance the readability, clarity, and professionalism of the manuscript. Thank you for your valuable guidance.

Conclusions

Reviewer 1

Reviewer comment 1: (Lines 253–256)

The authors clearly say improving knowledge and attitudes will strengthen Mpox control and preparedness in Sub-Saharan Africa.

Make the link broader, for example, explain that better-prepared healthcare workers could also improve early detection of future outbreaks, not only Mpox.

Author`s response: Thank you for your valuable suggestion to broaden the impact of our findings. As recommended, we have expanded the Conclusion to clarify that improving healthcare workers' knowledge and attitudes toward mpox will not only strengthen mpox control and preparedness in Sub-Saharan Africa, but also enhance early detection and response capabilities for future outbreaks of mpox and other emerging infectious diseases. This addition appears in the Conclusion section on page 17, lines 320–336. We appreciate your insightful guidance in strengthening the public health relevance of our work.

Editorial and Data Presentation Modifications

Reviewer 2

Reviewer 2 request: Correct formatting issues ("Error! Reference source not found") in text and figures.

Author`s response: we sincerely apologize for these reference errors. We have thoroughly checked and corrected all citations of figures and tables.

Reviewer 2 request: Ensure consistent terminology: “knowledge,” “good knowledge,” “positive attitudes,” and “favorable attitudes” should be harmonized.

Author`s response: we agree with the need for consistent terminology. We have carefully reviewed the manuscript and harmonized these terms, now consistently using good knowledge and positive attitudes through the entire manuscript.

Reviewer 3 request: Figures could benefit from higher resolution and uniform labeling.

Author`s response: thank you for this valuable suggestion. We have prepared the figures in better resolutions using the suggested tool (NAAS).

Reviewer 4 request: Minor language editing is needed to improve readability (some awkward phrasing).

Author`s response: we acknowledge this point and have conducted a thorough language and copy editing through the entire manuscript to improve readability and address awkward phrasing.

Summary and General Comments

Reviewer-1 (Weakness)

1. **Missing figures/tables with “Error! Reference source not found”**
   All figures and tables have been inserted, labeled correctly, and referenced throughout the manuscript.
2. **High heterogeneity (I^2 > 95%)**
   We acknowledge the high heterogeneity and have conducted additional sensitivity and meta-regression analyses. The limitations of pooled estimates are now explicitly discussed in the manuscript.
3. **Non-standardized definitions of “good knowledge” and “positive attitudes”**
   We have clarified outcome definitions in the Methods section and noted variability across studies as a limitation affecting comparability.
4. **Geographic bias (Nigeria and Ethiopia)**
   The over-representation of these countries is acknowledged as a limitation that may affect generalizability to the broader Sub-Saharan African region.

5. **Limited sensitivity/subgroup analyses**
   Additional analyses have been performed (meta-regression by publication year and sample size, sensitivity analysis). These are reported in the manuscript with interpretation of their findings.
6. **Incomplete/incorrect references**
   All references have been revised and formatted in full Vancouver style.
7. **Conclusions should better reflect limitations**
   The conclusion has been revised to explicitly remind readers of key study limitations, including high heterogeneity, language bias, and geographic concentration.

Thank you for your thorough and constructive feedback, which has significantly strengthened the manuscript

Reviewer 2 (Recommendation and required revision)

Response to Reviewer 2 comments

Thank you for your thorough and insightful review. We have carefully considered your suggestions and revised the manuscript to strengthen the introduction, discussion, and policy relevance. Below is a point-by-point summary of how we addressed each comment.

Introduction – Knowledge Gap and Rationale

- **Revised to clearly articulate the knowledge gap earlier in the introduction.** We now explicitly cite the previous global systematic reviews showing pooled estimates of 26–32% early in the introduction (lines 76–78), immediately after stating the general problem of suboptimal HCW knowledge and attitudes toward mpox.
- **Sharpened the rationale for focusing on Sub-Saharan Africa (SSA).** We have clarified why SSA differs from global patterns by highlighting factors such as endemicity of mpox, healthcare infrastructure challenges, regional disparities in training, and the disproportionate burden of cases in SSA following the 2022 outbreak.
- **Improved structure of the introduction.** The introduction now more clearly outlines:-
  What was known: Low global knowledge/attitudes among HCWs, especially in non-endemic regions pre-2022.

What remained uncertain: Whether post-2022 improvements occurred in SSA, and how knowledge and attitudes vary across SSA countries.

How this study fills the gap: By providing the first SSA-specific meta-analysis post-2022 to inform targeted, context-sensitive interventions.

- **Discussion – Reducing Repetition and Strengthening Policy Implications**
  - ✓ **Reduced repetitive mentions of heterogeneity.** Heterogeneity is now discussed concisely in one dedicated paragraph in the discussion, focusing on its sources and implications rather than reiterating statistical values.
  - ✓ **Expanded policy and program implications.** After discussing the finding of this meta-analysis, a paragraph has been added to discussion dealing about the program implications including developing and disseminating standardized, context-appropriate mpox training modules and integrating mpox education into pre-service and in-service HCW training.

Additional note on study inclusion during revision

During our revision, we successfully obtained one additional eligible study (Sofonias et al, 2024) that was initially inaccessible and not retrieved despite multiple database and author contact attempts. This study assessing mpox knowledge among HCW in Ethiopia (Addis Ababa), which meets all inclusion criteria has been incorporated into the final analysis to enhance comprehensiveness and minimize selection bias. Its inclusion did not substantially alter the pooled estimates or conclusions of the review. The study`s inclusion enhances the review's validity by ensuring a more complete evidence base. All relevant sections of the manuscript including the PRISMA diagram, results, tables, and figures have been updated accordingly.

We thank the reviewers and editors for their constructive guidance and suggestions which have significantly improve our manuscript. We believe these revisions have significantly strengthened the manuscript's rationale, clarity, and practical relevance. We are grateful for the insightful feedback and hope the revised manuscript now meets the journal`s standards for publication.

We look forward to your assessment of the revised manuscript and welcome any further feedback

Melaku Laikemariam

Corresponding author

On behalf of all authors
