# Supplementary material for: Mpox knowledge and positive attitudes in Sub-Saharan African healthcare workers after 2022 outbreak of disease: A systematic review and meta-analysis
Source: PLoS Negl Trop Dis. 2026 Feb 9;20(2):e0013977. doi: 10.1371/journal.pntd.0013977 (PMC12900440; doi:10.1371/journal.pntd.0013977)
Supplement: Attachment — Submitted filename: Response_to_Reviewers_auresp_2.pdf [file pntd.0013977.s008.pdf]

Response to Reviewers

Manuscript ID: PNTD-D-25-01469R1

Title: Mpox Knowledge and Positive Attitudes in Sub-Saharan African Healthcare Workers After 2022 Outbreak of Disease: A Systematic Review and Meta-Analysis.

Journal: PLOS Neglected Tropical Diseases

Dear Editors and Reviewers,

Thank you for the opportunity to revise and resubmit our manuscript. We sincerely appreciate the thoughtful and constructive feedback provided by the editors and reviewers, which has significantly strengthened our work. We have carefully addressed each comment and have incorporated all suggested revisions. Below, we provide a point-by-point response to each comment, detailing the changes made in the manuscript. We believe these revisions have significantly strengthened the manuscript, and we are grateful for the insightful guidance.

Editor`s comment 1: Provide a short title for the manuscript according to the journal guidelines

Author`s response: We sincerely thank the Editor for the important guidance regarding the provision of a short title in accordance with the journal’s formatting requirements. In direct response to this instruction, we have added the short title **“Mpox Knowledge and Positive Attitudes in Sub-Saharan African Healthcare Workers”** to the title page of the manuscript, positioning it after the full title and before the author list. This has also been entered into the designated field in the submission system to ensure proper formatting. We are grateful for this valuable and constructive editorial direction, which has enhanced the presentation of our manuscript.

Editor`s comment 2: What is the rationale for specifying a 50% threshold in the hypothesis? This value appears arbitrary and falls below commonly accepted benchmarks for “good” knowledge or positive attitudes in KAP studies. Please justify the choice of this cut-off or consider reframing the hypothesis without a fixed threshold.

Author`s response: We thank the Editor for the critical observation regarding the 50% threshold in our initial hypothesis. We fully agree that this value was arbitrary and did not align with standard benchmarks for knowledge, attitude, and practice (KAP) studies. In response to your insightful suggestion, we have reframed the hypothesis to be conceptually driven rather than anchored to an arbitrary numerical cutoff. The revised hypothesis now states: "**This review hypothesized that the pooled proportion of healthcare workers with good mpox knowledge and positive attitudes would be below optimal levels, reflecting significant gaps in awareness and preparedness in Sub-Saharan Africa despite the 2022 outbreak**."

This modification shifts the focus to a qualitative expectation of suboptimal preparedness, which is better justified by the prior evidence of low global estimates and regional challenges cited in our introduction. We believe this revision strengthens the scientific rigor and clarity of our study's framework. We are grateful for this constructive feedback, which has significantly improved our manuscript.

Editor's comment 3: Please revise the formatting of Figure 1 (PRISMA flow diagram). Several arrows appear distorted, and the boxes are not properly aligned. Ensure consistent box sizes, uniform arrow styles, and clear alignment so that the figure is visually coherent and easy to follow.

Author`s response: Thank you for your detailed feedback regarding the formatting of Figure 1 (PRISMA flow diagram). We recognize that the visual clarity of this figure is paramount for

accurately conveying the study selection process. In direct response to your guidance, we have completely recreated the PRISMA diagram using specialized software tools to ensure professional alignment and visual coherence. The updated figure now features consistently sized boxes, uniform arrow styles, and clear, logical alignment, making the flow of study identification, screening, and inclusion fully transparent and easy to follow. We are grateful for this constructive suggestion, which has significantly enhanced the presentation of our methodological workflow.

Methods

Reviewer 2 comment #1: The objectives of the study are clearly articulated and aligned with the stated aim of synthesizing evidence on mpox-related knowledge and attitudes among healthcare workers in Sub-Saharan Africa following the 2022 outbreak. While an explicit testable hypothesis is not formally stated, this is acceptable given the systematic review and meta-analysis design.

Author`s response: We thank Reviewer #2 for their positive evaluation that the "objectives of the study are clearly articulated and aligned with the stated aim." We also appreciate their acceptance of the systematic review design, where a formal testable hypothesis is not always mandatory. However, following the valuable editorial guidance, we have taken the opportunity to enhance the study's conceptual framework. We have now articulated a more focused, guiding hypothesis within the Introduction. The revised hypothesis states that the pooled proportion of healthcare workers with good knowledge and positive attitudes would be below optimal levels, reflecting significant preparedness gaps despite the 2022 outbreak. This addition provides clearer direction for the synthesis while remaining appropriate for the meta-analytic design.

Results

Reviewer 1 comment #1: The analysis strictly followed the pre-specified Statistical Analysis Plan (SAP). The PALM 007 trial (NCT05559099) was designed as a double-blind, randomized, placebo-controlled trial. The researchers adhered to the protocol by focusing on the primary endpoint time to complete resolution of all lesions.

Author`s response: We thank you for your positive assessment of the study's methodological rigor. We acknowledge that part of their feedback references a specific clinical trial context ("PALM 007 trial), which does not apply to our systematic review and meta-analysis design. Our study synthesizes observational data on healthcare workers' knowledge and attitudes, and we did not pre-specify or follow a clinical trial Statistical Analysis Plan (SAP). We confirm that our analysis strictly adhered to the pre-registered systematic review protocol and the analysis plan detailed in our Methods section, which included a random-effects meta-analysis, assessment of heterogeneity, subgroup analysis, and publication bias assessment as appropriate for the included observational studies. We appreciate the reviewer's recognition of our adherence to our stated methodological approach.

Reviewer 2 comment #1: The analyses presented are consistent with the stated analysis plan. Results are clearly structured and logically presented, with appropriate use of tables and figures to summarize pooled estimates and heterogeneity measures.

The figures and tables are of acceptable quality and enhance clarity and interpretability of the findings. Minor improvements in figure labeling or caption clarity could further enhance readability, but no substantive issues are identified.

Author`s response: We thank Reviewer #2 for their positive assessment that our analyses align with the stated plan, that the results are clearly presented, and that the figures and tables are of acceptable quality. We are also grateful for the constructive suggestion to improve readability through enhanced labeling and captions. We sincerely appreciate this valuable feedback.

Conclusion

Reviewer 1 comment #2: The conclusions are rigorously grounded in the data, even where they challenge initial hypotheses.

Author`s response: We thank Reviewer #1 for their positive assessment that our "conclusions are rigorously grounded in the data." We are particularly encouraged by the acknowledgment that our interpretations remain robust even where they may challenge initial expectations. In alignment with this feedback and the editorial guidance, we have revised the study's hypothesis to be conceptually driven, removing the specific numerical threshold. This refinement strengthens the framework of our inquiry. We thank you for your valuable guidance and suggestions.

Editorial and Data Presentation Modifications

Reviewer 1 comment #1: The manuscript is scientifically sound and addresses a critical public health gap. The recommendation for "Minor Revision" is based on the need for the authors to more explicitly frame the "Standard of Care" finding as a central success of the trial, ensuring clinicians do not walk away with the simple (and potentially incorrect) message that "the drug doesn't work" without understanding the context of the care provided.

Author`s response: We thank Reviewer 1 for their positive assessment that our manuscript is 'scientifically sound and addresses a critical public health gap. We note that the latter part of their comment references clinical trial findings ('Standard of Care,' 'the drug doesn't work'), which do not apply to our systematic review and meta-analysis. Our study synthesizes observational data on healthcare workers' knowledge and attitudes, not clinical trial outcomes. We have ensured that our conclusions appropriately frame our findings within the context of public health preparedness and training needs.

Reviewer 2 comment #1: Only minor editorial refinements are suggested, such as ensuring consistent terminology across sections and clarifying figure captions where appropriate. No additional analyses or major data modifications are required.

Author`s response: We sincerely thank the Reviewer for your insightful suggestions and guidance. We have ensured consistency of terminology across the entire manuscript, and some amendments were performed on figure and table captions to enhance their clarity for readers. We are grateful for the constructive suggestion to further enhance readability through improved figure labeling and captions.

We thank the reviewers and editors for their constructive guidance and suggestions, which have significantly improved our manuscript. We believe these revisions have significantly strengthened the manuscript's rationale, clarity, and practical relevance. We are grateful for the insightful feedback and hope the revised manuscript now meets the journal`s standards for publication.

We look forward to your assessment of the revised manuscript and welcome any further feedback

Melaku Laikemariam

Corresponding author

On behalf of all authors
